# Proliferation of nutrition sensing preadipocytes upon short term HFD feeding

**DOI:** 10.1080/21623945.2018.1521229

**Published:** 2018-09-29

**Authors:** Elisabeth Kulenkampff, Christian Wolfrum

**Affiliations:** Institute of Food Nutrition and Health, Eidgenössische Technische Hochschule Zürich (ETH), Schwerzenbach, Switzerland

**Keywords:** Epididymal adipose tissue, hyperplasia, hypertrophy, preadipocytes, nutrition sensing, proliferation and age, high fat diet (HFD)

## Abstract

Adipose tissue is highly dynamic and increases its size dependent on the status of nutrition. Generally, an increase of adipose tissue mass is attributed to two mechanisms, namely hypertrophy (increase in adipocyte size) and hyperplasia (increase in adipocyte number). Here, we analyzed the proliferation capacity of a pool of nutrition sensing preadipocytes after short-term high fat diet (HFD) feeding. We show that this process is age independent and that adipocyte hyperplasia seems not to be dependent on adipocyte hypertrophy. Further, we could show that the subsequent development into adipocytes is influenced by the duration of HFD feeding after proliferation. Our data also demonstrate that the studied pool of preadipocytes seems to be finite and cannot be reactivated by multiple bouts of HFD feeding. In conclusion, our results indicate an important link between stem cells, nutrition status and homeostasis in the epididymal adipose tissue.

## Introduction

Adipose tissue is one of the major endocrine organs and plays an important role in the metabolic homeostasis of an organism.^1^ It is located in various areas of the body where it functions both in distinct and overlapping capacities. Adipose tissue consists from a volumetric point of view mainly of adipocytes, however immune cells, fibroblasts, endothelial cells and preadipocytes are also found within the adipose tissue stromal vascular fraction (SVF).

The external supply of energy to an organism is in general not continuous, therefore, energy has to be stored and released based on the availability of external fuel sources. In this context, adipose tissue shows a highly dynamic behavior. For example, it can increase in size very rapidly in response to an overload of energy. Generally, an increase of adipose tissue mass is attributed to two mechanisms: hypertrophy (increase in adipocyte size) and hyperplasia (increase in adipocyte number). It was shown that in as short as 24h of fasting and refeeding, changes in cell size and gene expression were detectable in various adipose tissue depots.^2,3^ Long- and short-term overnutrition has been shown to directly influence adipose tissue and to cause hypertrophy, inflammation, insulin resistance, and changes in plasma parameters in mice and humans.^2,4–9^ Conversely, removal of HFD was shown to reverse most of these changes.^2,10,11^

For some time it was believed, that the total number of fat cells is set during adolescence and a change of fat mass in adults is exclusively caused by hypertrophy.^12,13^ However, it was recently demonstrated that hyperplasia plays an important role in obesity and that the ability for an increase in adipocyte number persists for lifetime even after severe weight loss.^14–16^ In obesity there is a balance between hyperplasia and hypertrophy, which influences the metabolic outcome of obesity.^17^ Various studies have shown that hyperplasia in visceral adipose tissue is advantageous compared to hypertrophy.^18,19^ In addition, it was shown that an increasing of number of healthy adipocytes in visceral adipose tissue correlates with insulin sensitivity in obese patients.^9,20^

Mature adipocytes are post-mitotic; thus, hyperplasia requires the existence of preadipocytes, which proliferate and differentiate into mature adipocytes. In several studies, it was shown that preadipocytes are found in the SVF usually in the perivascular region.^21–23^ Various markers were proposed to define preadipocytes, however the exact population of proliferative cells and the timing of preadipocyte activation for division, as well as the underlying molecular cues are still not known.^24^ One population generally accepted to contain adipocyte precursor cells are Lin-PDGFRa+ cells, which are able to give rise to brite and white adipocytes.^25,26^

Recent studies have reported that certain external stimuli are able to increase the number of proliferative preadipocytes in specific depots.^24,27^ After high fat diet (HFD), subcutaneous adipose tissue (SUB) was suggested to increase in size by hypertrophy while visceral adipose tissue (VIS) size increase is at least in part due to adipocyte hyperplasia.^24^ This is due to an HFD-mediated induction of preadipocyte proliferation followed by activated adipogenesis over several weeks.^24^

Distribution and cellularity of adipose tissue changes generally with age and each fat depot is affected differently by aging.^28–31^ It was reported that the abundance of preadipocytes and reaction to HFD when started directly after weaning (3 weeks) is highly increased compared to 8 week old mice.^32^ This indicates a high dynamic in visceral adipose tissue especially at the developmental phase of the tissue.

Other studies showed that further substantial changes in adipose tissue prevail between middle and old age.^33^ Body fat mass peaks at middle age and declines with old age. In addition, a redistribution from subcutaneous to visceral adipose tissue has been observed in the elderly, which is accompanied by an increased risk of age-related diseases and obesity.^31,34^

## Results

### Age dependent altered reaction of adipose tissue to overnutrition

**Figure 1.**
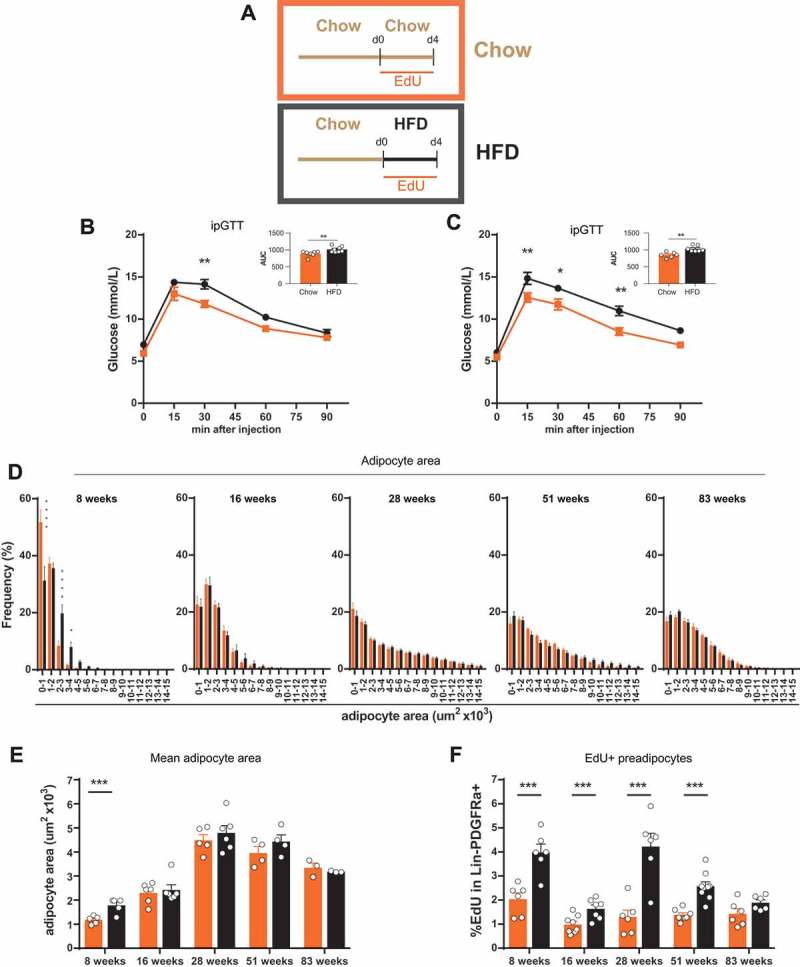
**Age dependent altered reaction of adipose tissue to overnutrition. (A)** Experimental scheme for feeding and treatment strategy. Different age groups of male mice (young (8 weeks), adult (16 weeks), middle aged (28 weeks), old (51 weeks) and very old (83 weeks)) were fed 4 days with HFD or chow diet in combination with EdU treatment to label proliferating preadipocytes in eWAT. (**B-C)** Intraperitoneal glucose tolerance test in **(B)** young (8 week) (chow n = 7; HFD n = 8) and **(C)** old (51 week) mice (chow n = 6; HFD n = 7). **(D)** Frequency distribution of adipocyte area and **(E)** mean adipocyte area in eWAT (8 weeks n = 5; 16 weeks n = 6; 28 weeks (chow n = 5; HFD n = 6); 51 weeks n = 4; 83 weeks n = 3; per group). **(F)** Quantification of EdU incorporation in preadipocytes (Lin-PDGFRa+) in eWAT (8 weeks n = 6; 16 weeks (chow n = 8; HFD n = 7); 28 weeks n = 6; 51 weeks (chow n = 7; HFD n = 9); 83 weeks n = 6). Error bars represent mean ± s.e.m. *(P < 0.05), **(P < 0.01), ***(P < 0.001), **** (P < 0.0001), by Student`s t-test **(B-C and E-F)**, 2-way ANOVA + Bonferroni post hoc analysis **(D)**. If not indicated otherwise n denotes individual mice. eWAT, epididymal adipose tissue; HFD, high fat diet; EdU, 5-Ethynyl-2ʹ-deoxyuridine.

Changes in metabolism, distribution and cellularity of adipose tissue is dependent on intrinsic factors such as age, as well as extrinsic factors such as overnutrition.^2,28^ We aimed to study how HFD affects adipose tissue dynamics in relation to age. To this effect, we fed mice of various ages (young (8 weeks), adult (16 weeks), middle aged (28 weeks), old (51 weeks) and very old (83 weeks)) chow or HFD for 4 days and labeled proliferating preadipocytes by administering EdU in the drinking water (Schema in Figure 1A). Analysis of glucose tolerance in young (Figure 1B) and old (Figure 1C) mice demonstrated that short-term HFD treatment was sufficient to impair glucose tolerance independent of age. Interestingly, we did not observe any differences in baseline glucose tolerance and HFD-induced glucose intolerance in relation to age with the exception of a slightly slower normalization to baseline at 60min after injection in old mice (Figure 1C).

It has been reported that the weight of visceral adipose tissue increases during maturation until middle age and declines in old/senescent rats without any stimulus.^35,36^ Therefore, we measured the adipocyte cell area frequency distribution in epididymal adipose tissue after 4 days of HFD or chow feeding in the five age groups. We observed that the overall adipocyte area increased with age up to middle age (28 weeks), remained stable until old age (51 weeks) and decreased in very old mice (83 weeks) (Figure 1D-E). These values also correlate with the weight of the mice and the epididymal adipose tissue weight (Sup. Figure 1B-C). A significant distribution shift to bigger adipocytes (hypertrophy) upon HFD feeding was only detected in the youngest mice (8 weeks) tested (Figure 1D-E). This cannot be attributed to calorie intake, because the youngest mice tested did not change their calorie intake, whereas old mice showed slightly increased calorie intake (Sup.Figure 1D).

It was recently reported that short-term (3–4 days) as well as long-term HFD feeding result in an increase in preadipocyte proliferation in young (6–8 week) mice.^24,37^ Given that we only observe a hypertrophic response in mice of this particular age group, we wanted to investigate whether a link exists between adipocyte hypertrophy and the onset of preadipocytes proliferation and whether the capacity to induce adipogenesis by HFD is lost during the aging process of mice.

We measured proliferation capacity by EdU incorporation into Lin(CD31,CD45,Ter119)-PDGFRa+ preadipocytes (Figure 1F and gating strategy Sup. Figure 1A). All age groups showed a baseline proliferation rate of about 1–2%. Upon HFD feeding, we observed a significant increase of EdU labeled cells (about 2–4%) in all groups except for the very old mice, which did not show a significant change in proliferation from baseline.

Taken together, our results show that impairment of glucose tolerance upon short HFD treatment is age independent. HFD-induced hypertrophy was only detectable in young mice, possibly due to the average adipocyte size. HFD-induced proliferation of Lin-PDGFRa+ preadipocytes, on the other hand, occurs in young to old mice and seems therefore to be independent of adipocyte hypertrophy.

### Activated preadipocytes undergo adipogenesis

**Figure 2.**
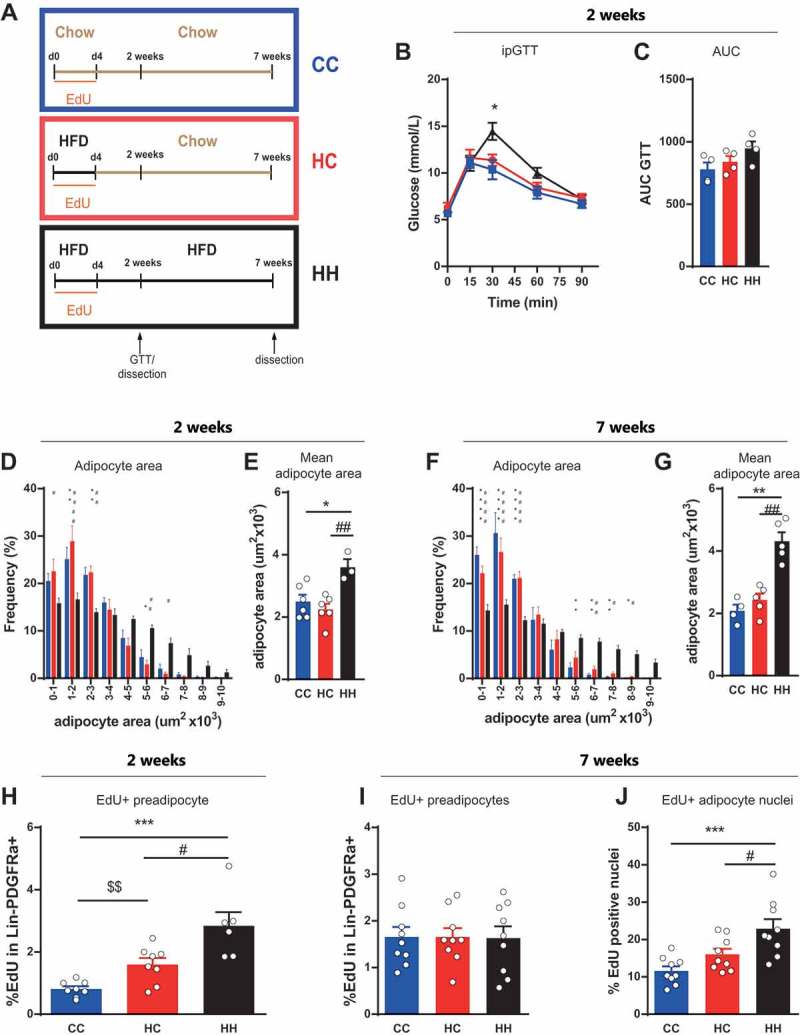
**Adipogenesis of activated preadipocytes. (A**) Feeding and treatment strategy. Mice were fed with HFD (HC and HH) or chow (CC) in combination with EdU treatment for 4 days (dietary intervention). They were subsequently fed chow (HC, CC) or HFD (HH) for 2 or 7 weeks without any further EdU treatment. (**B)** Intraperitoneal glucose tolerance test and **(C)** corresponding area under the curve (AUC) 2 weeks after dietary intervention (n = 4 per group). **(D-G)** Frequency distribution of adipocyte area and mean adipocyte area in eWAT depot **(D-E)** 2 weeks (CC and HC n = 6; HH n = 3) and **(F-G)** 7 weeks (CC n = 4; HC and HH n = 5) after dietary intervention. **(H-J)** Quantification of EdU incorporation into **(H-I)** preadipocytes or **(J)** adipocyte nuclei in eWAT after **(H)** 2 weeks (CC and HC n = 8; HH n = 6) or **(I-J)** 7 weeks (n = 9 per group) dietary intervention. Error bars represent mean ± s.e.m. *(P < 0.05), **(P < 0.01), ***(P < 0.001) by 2-way ANOVA + Tukey’s multiple comparison. * = CC vs HH; $ = CC vs HC; # = HC vs HH. Unless indicated otherwise, n denotes individual mice. eWAT, epididymal adipose tissue; HFD, high fat diet; EdU, 5-Ethynyl-2ʹ-deoxyuridine.

Based on our findings that proliferation of preadipocytes and glucose tolerance in response to a HFD challenge are triggered independently of age, we aimed to investigate whether a prolonged HFD challenge and consequent prolonged glucose intolerance would induce the recruitment of additional adipocytes. To study this we fed young mice for 4 days with HFD (HC and HH) or chow (CC) and administrated EdU in the drinking water. The mice where subsequently fed chow (HC, CC) or HFD (HH) for 2 or 7 weeks without any further EdU treatment (Scheme Figure 2A).

In a first experiment, we analyzed whether reduced glucose tolerance is acutely triggered by HFD treatment or whether short HFD feeding is sufficient to induce long lasting changes in glucose tolerance. Analysis of glucose tolerance two weeks after diet change did not demonstrate any difference between the groups fed with chow (HC and CC) (Figure 2B-C). In contrast, we observed impaired glucose tolerance in the HH group, which was fed HFD for the entire time of the experiment. These data demonstrate that glucose tolerance is acutely influenced by the nutritional status but quickly reverted upon removal of the HFD stimulus. Adipocyte area distribution and mean adipocyte cell area showed no difference between the chow fed groups (CC and HC) after 2 or 7 weeks (Figure 2D-G). In contrast, the HFD group (HH) showed comparatively elevated adipocyte area at both time points. These results demonstrate that adipose tissue reacts and reverts rapidly to the status of nutrition to be stored.

To address whether the nutrition status influences the activated preadipocytes after the initial 4 days of labeling and the formation of new adipocytes, we analyzed EdU incorporation into Lin-PDGFRa+ preadipocytes and adipocyte nuclei in these mice. Our results show that after 2 weeks on chow the amount of EdU labeled preadipocytes differs significantly among all three groups (Figure 2H). The amount of EdU labeled cells increased in both groups initially fed with HFD (HC and HH) as compared to the chow (CC) group (Figure 2H), indicating that the proliferation was directly triggered by the short HFD as expected from our earlier results. Surprisingly, the number of EdU positive preadipocytes exhibits a significant difference in HC vs HH with a much higher number in the group continued on HFD (HH) (Figure 2H). After 7 weeks, a similar difference in EdU incorporation was observed in the adipocyte nuclear fraction (Figure 2J), whereas the amount of EdU labeled preadipocytes at this time showed equal amounts in all three treatment groups (Figure 2I).

Taken together, our data indicate that continuous HFD challenge concomitant with prolonged glucose intolerance has an influence on the activated preadipocytes after the initial 4 days of labeling since the number of labeled preadipocytes after 2 weeks and adipocytes after 7 weeks, depended on the duration of the HFD feeding.

### Reaction of epididymal adipose tissue to a second short term HFD feeding

Our results imply that activated preadipocytes transition into an adipocyte lineage committed cell type and continue to undergo adipogenesis independently of the nutritional status. We were further interested in investigating the dynamics of this pool of preadipocytes and whether we are able to reactivate them after an intermediate period of chow feeding.

**Figure 3.**
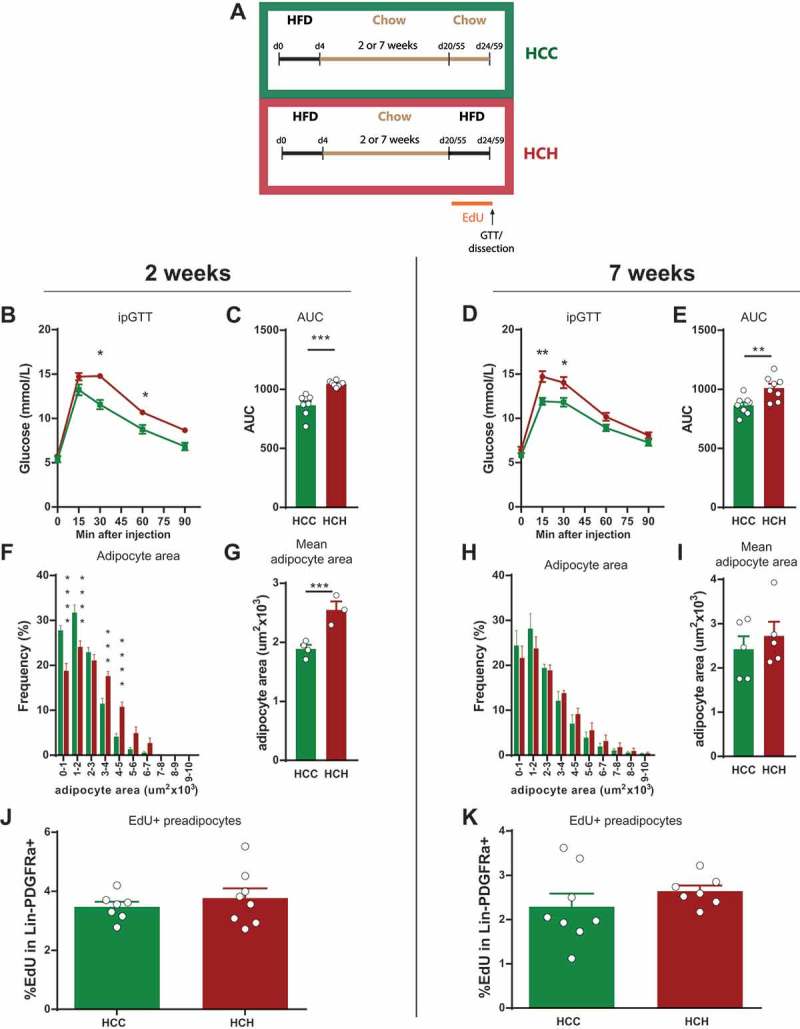
**Reaction of epididymal adipose tissue to a second short term HFD feeding. (A)** Experimental scheme for feeding and treatment strategy. Mice were fed HFD for 4 days followed by a chow diet (intermediate feeding) for 2 or 7 weeks. This was followed by a second dietary intervention (refeeding) with chow (HCC) or HFD (HCH) in combination with EdU treatment for 4 days. (**B-E)** Intraperitoneal glucose tolerance test and corresponding AUC (2 weeks (HCC n = 7; HCH n = 8); 7 weeks (n = 8 per group). **(F-I)** Frequency distribution of adipocyte area and mean adipocyte area in eWAT after refeeding (2 weeks (HCC n = 4; HCH n = 3); 7 weeks (n = 5 per group)). **(J-K)** Quantification of EdU incorporation in preadipocytes (Lin-PDGFRa+) in eWAT after refeeding (2 weeks (n = 7 per group); 7 weeks (HCC n = 8; HCH n = 7). Error bars represent mean ± s.e.m. *(P < 0.05), **(P < 0.01), ***(P < 0.001), **** (P < 0.0001), by Student`s t-test **(B-E, G, I, and J-K)**, 2-way ANOVA + Bonferroni post hoc analysis **(F and H)**. Unless indicated otherwise, n denotes individual mice. eWAT, epididymal adipose tissue; HFD, high fat diet; EdU, 5-Ethynyl-2ʹ-deoxyuridine; AUC, area under curve.

We fed mice HFD for 4 days followed by a chow diet for 2 or 7 weeks. Subsequently, one group of mice was re-exposed to HFD (HCH) while the other group was kept on chow diet (HCC) for another 4 days. Proliferating cells were labeled by administration of EdU in the drinking water during the second HFD/Chow feeding (Schema Figure 3A). Glucose tolerance was impaired after the second HFD treatment independently of chow duration (Figure 3B-E) showing a direct link between nutrition status and glucose intolerance. Also, adipocyte hypertrophy upon HFD re-feeding was triggered when mice were fed 2 weeks of intermediate chow diet (Figure 3F-G) while this could not be detected after 7 weeks of intermediate chow feeding (Figure 3H-I). We assume that this is not directly correlated with the re-exposure to HFD, but age dependent, as suggested from Figure 1D-E. After 7 weeks on chow the mice are around 15 weeks old and show no hypertrophy and the adipocyte area distribution correlates with the adult mice (16weeks) in Figure 1D-E.

In contrast, proliferation seems to be impaired after re-exposure to HFD as we could not detect any increase in EdU incorporation in the Lin-PDGFRa+ pool upon re-exposure to HFD as compared to chow treated animals (Figure 3J-K). This observation was also independent of the length of the intermediate chow period. From these results we would speculate that the studied pool within the Lin-PDGFRa+ lineage is finite and is not replenished.

## Discussion

Hypertrophy was long assumed to stimulate the activation and proliferation of preadipocytes when the maximal size capacity of adipocytes is reached.^15,38^ Our results suggest that there is no such hierarchical process, since adipogenesis is rapidly initiated and long before the existing adipocytes are assumed to reach their maximal size capacity. Hypertrophy which is detectable only in young mice after HFD exposure reappears after refeeding, whereas preadipocyte proliferation is detectable up to old age, but restricted to first dietary intervention. We therefore postulate that hypertrophy is not the driver for hyperplasia, but that these processes are independent from each other.

Earlier studies already described age-related changes in fat tissue function and cellularity.^36^ We show here that proliferation of preadipocytes after HFD feeding occurs up to old age; only in very old mice (83 weeks) we could not detect a substantial increase of this process. We conclude from these findings that the here studied pool of proliferative preadipocytes persists and is activatable throughout life. In recent years, various markers of different developmental origins e.g. vascularity were proposed to specify the preadipocytes lineage.^39^ To our knowledge, a “perfect” marker to define preadipocytes is still not found. We therefore used a preadipocyte population which is defined by Lin-PDGFRa+ expression. This population is generally accepted to give rise to white and brite cells, but it cannot be excluded that other subpopulations of preadipocytes exist.

It was suggested before that hyperplasia promotes insulin sensitivity contrary to hypertrophy.^40^ This might suggest that the capability of epididymal adipose tissue to react to overnutrition by inducing hyperplasia up to an old age allows the storage of lipids in a healthier manner. Unfortunately, a direct comparison of the degree of proliferation dependent on age is not possible since EdU was stained after fixation of the cells and therefore a bias in staining intensity at different days might influence the percentage of baseline proliferation among all measured age groups.

Concerning the dynamics of the induction of proliferation of preadipocytes and their subsequent development into adipocytes, experiments from an earlier study are based on continuous feeding with HFD.^24^ The study reported highest proliferation of preadipocytes in response to HFD after 3 days and return to chow levels after 5 days. It was furthermore shown that these cells develop into lipid-filled adipocytes after 7 weeks of continuous HFD feeding. In order to obtain a better understanding of these processes, we used a scheme of variable feeding and observed a significant difference in EdU-labeled cells between continuous HFD feeding and the group that was switched to a chow diet. Our findings thus indicate that nutrition status continues to influence the proliferating preadipocytes and their subsequent development into adipocytes. Since the labeling with EdU was terminated after 4 days in all tested groups, it is unlikely that new cells are recruited for proliferation. However, it cannot be excluded that a low percentage of mature adipocytes proliferate in this timeframe or that immune cells attached to the adipocytes contribute to the pool of EdU-labeled nuclei. From these results we propose two possible mechanisms which would need further experimental evaluation: (1) The pool of cells proliferating during the first 4 days undergo another round of proliferation when the mice are kept on HFD to increase the pool of preadipocytes. In cell culture systems, it has been well established that preadipocytes undergo mitotic clonal expansion before they develop into adipocytes.^41^ It is therefore possible that a similar process is occurring *in vivo*. (2) Since cells appear to be dependent on nutrient sensing, they might rapidly undergo apoptosis when the trigger of HFD is removed and no further increase in adipocyte number is required. Further tests are required to investigate whether one of these scenarios is the explanation for the observed phenotype.

A turnover of adipocytes without external stimuli was shown to occur at any time to maintain functional adipose tissue.^16^ Since this process is conserved throughout life, the pool of preadipocytes is assumed to be replenished after proliferation. Our results indicate that HFD-induced proliferation of preadipocytes is restricted to naïve mice and does not reoccur after HFD refeeding. Based on these results, we speculate that a distinguishable pool of preadipocytes is responsible for this process and that preadipocytes transition into a committed cell type after proliferation and lose their capability for self-renewal. This might be a regulatory mechanism of the adipose tissue to control for adipocyte number, but needs further experimental validation.

In summary, we propose that a specific pool of nutrition sensing preadipocytes exists in the epididymal adipose tissue. This pool is kept during life to potentially enable the tissue to react rapidly to overload of nutrition. The nutrition status is important for the early commitment and subsequent adipocyte formation of these cells underscoring an important link between stem cells, nutrition status and homeostasis in the epididymal adipose tissue.

## Methods

### Mouse

C57BL/6N male mice (2–3 per IVC cage) were housed at an ambient temperature of 23°C with an inverted 12-h light/dark cycle and free access to food and water. Mice were fed standard chow (Kliba-Nafag purified diet #2222; 18% protein, 7% fat, 58% NFE) or a 60% calories high fat diet (Kliba-Nafag purified diet #2127; 23.9% protein, 35% fat, 23.2% NFE) (NFE: Nitrogen-free extract, containing carbohydrates, sugars and starches”) for the described times. Unless otherwise noted, mice were 7–9 weeks of age at the beginning of the experiments.

Before changing to HFD, mice were starved for 3h. EdU (5-ethynyl-2ʹ-deoxyuridine, Thermo Fisher, E10187) treatment was administered in drinking water (0.7mg/ml) for 4 days. All animal procedures were approved by national and institutional guidelines.

### SVF and adipocyte isolation

For SVF (stromal vascular fraction) and adipocyte isolation, epididymal adipose tissue was prepared as previously described.^42–44^ After separation, adipocytes and SVF were treated as follows:

Adipocytes were transferred into KRHB (50mM HEPES, 137mM NaCl, 4.7mM KCl, 1.85mM CaCl, 1.3mM MgSO4, 4mM Glucose, pH = 7.4), filtered through a 200um sieve and centrifuged for 1min at 150g. Adipocyte nuclei isolation was performed as previously described.^44^ In short, KRHB buffer was aspirated from under the adipocytes, leaving 1-2ml buffer. 3-4ml 0.4%IGEPAL in KRHB was added, vortexed for 1min and kept on ice for 10min with short shaking intervals. Samples were centrifuged at 2000g for 5min and washed with KRHB+ 0.002%IGEPAL. Nuclei were fixed in Click-iT fixative from the Click-iT™ EdU Alexa Fluor™ 488 Flow Cytometry Assay Kit (Invitrogen) for 15min at RT. EdU staining was performed using Click-iT™ EdU Alexa Fluor™ 488 Flow Cytometry Assay Kit (Invitrogen) according to the manufacturer’s instructions. Nuclei were diluted in FACS buffer (PBS, 3% FBS, 1% P/S, 1mM EDTA). 5ug/ml Hoechst was added and FACS analysis was performed on a MAQSQuant Analyzer 10.

SVF fraction were washed in DMEM and filtered through a 40μM cell strainer. Erythrocyte lysis was performed by washing the cells for 4min in erythrocyte lysis buffer (154 mM NH4Cl, 10 mM KHCO3, 0.1mM EDTA) at RT for 5min. Surface antigens (CD45-APCCy7, CD31-PECy7, Ter119-PECy7, PDGFRa-PE all BioLegend) were stained for 15min on ice. Live/Dead staining was performed using L/D fixable aqua (Thermofisher). Cells were fixed and EdU staining was performed using Click-iT™ EdU Alexa Fluor™ 488 Flow Cytometry Assay Kit (Invitrogen) according to the manufacturer’s instructions. Cells were diluted in FACS buffer (PBS, 3% FBS, 1% P/S, 1mM EDTA) and FACS analysis was performed on MAQSQuant Analyzer 10.

### Adipocyte size and frequency distribution analysis

Epididymal adipose tissues were excised and fixed in 4% paraformaldehyde for 48h. Processing was performed in an STP 120 Spin Tissue Processor (Thermoscientific) according to the standard protocol. Samples were embedded in paraffin and cut into 5 μm thin sections. Sections were de-paraffinized and stained with haematoxylin and eosin. Pictures were taken on Slide Scanner Pannoramic 250 (3D Histech) using an automated program. Pictures were analyzed with FIJI using a macro to determine adipocyte distribution and size. In average 4000 cells/mouse were analyzed.

### Macro

Macro adapted from.^45^

run(“Properties…”, “channels = 1 slices = 1 frames = 1 unit = pixel pixel_width = … pixel_height = … voxel_depth = …”); run(“Split Channels”); close(); close(); run(“Enhance Local Contrast (CLAHE)”, “blocksize = 127 histogram = 256 maximum = 3 mask = *None*”); run(“Invert LUT”); setThreshold(0, 81); run(“Convert to Mask”); run(“Remove Outliers…”, “radius = 2 threshold = 100 which = Bright”); run(“Dilate”); run(“Watershed”); run(“Analyze Particles…”, “size = 350–20,000 show = [Overlay Masks] display exclude”);

### Glucose tolerance test

Mice were fasted for 6h and injected intra-peritoneally (ip) with 1g/kg body weight Glucose in 0.9% NaCl. Blood was collected from the tail vein and blood glucose concentration was measured with a Glucometer (Accu-Check Aviva, Roche Diagnostics) in the described time intervals.

### Statistical analysis

Results are given as mean ± standard error of the mean (SEM). Statistical analyses were performed using two-tailed Student’s *t*-test, 1-way ANOVA or 2-way ANOVA as indicated. Significance was accepted at a value of P < 0.05.
