# Supplementary material for: Proliferation of nutrition sensing preadipocytes upon short term HFD feeding
Source: Adipocyte. 2018 Sep 29;8(1):16–25. doi: 10.1080/21623945.2018.1521229 (PMC6768278; doi:10.1080/21623945.2018.1521229)
Supplement: Supplemental Material [file kadi-08-01-1521229-s001.docx]

**
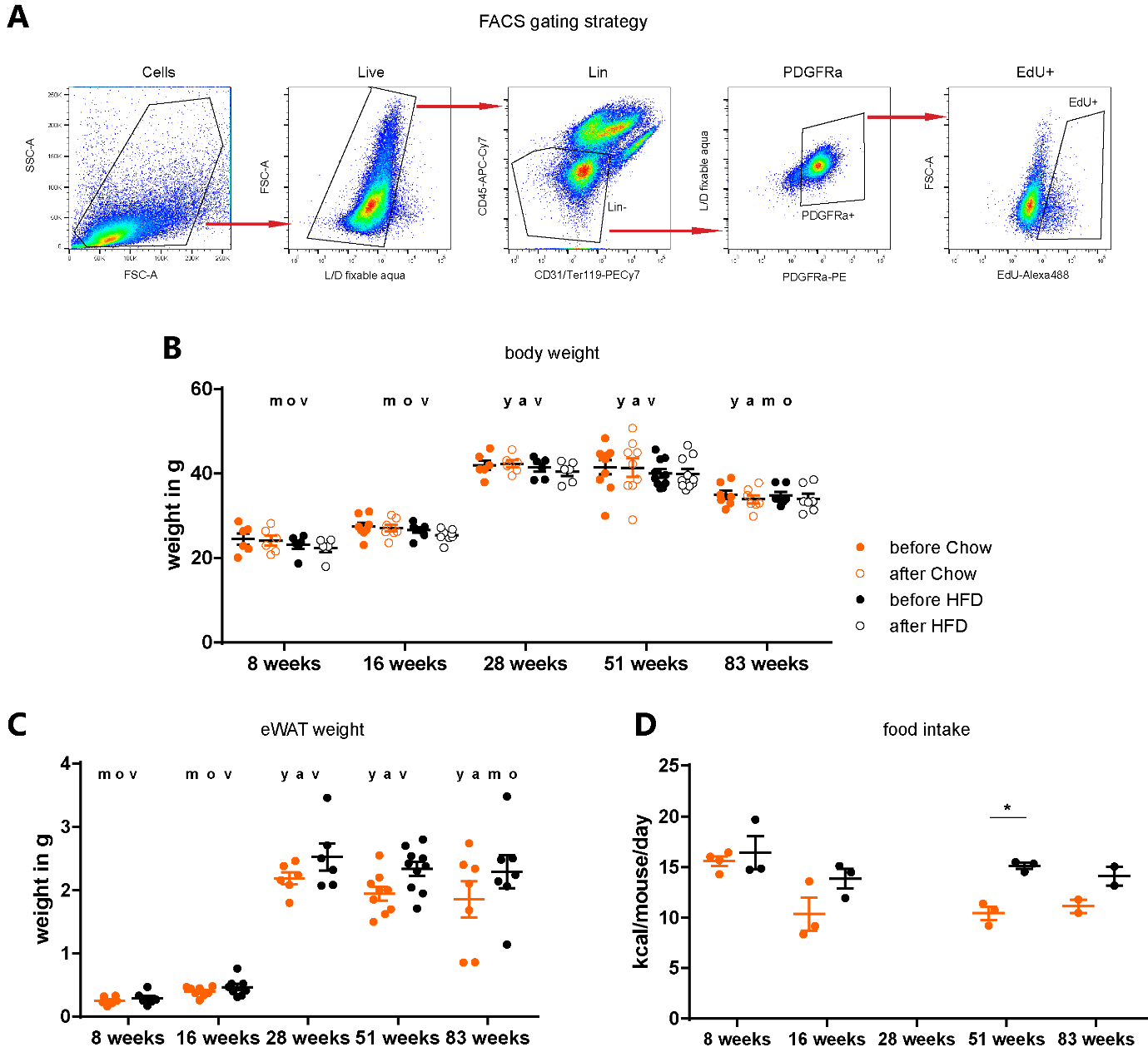
**

**Supplement figure 1**

**(A)** Representative flow cytometry dot blots showing sequential gating strategy used to identify proliferating (EdU+) preadipocytes (Lin-PDGFRa+). **(B)** Body weights of chow (orange) and HFD (black) before (solid) and after (open) dietary intervention and **(C)** corresponding eWAT tissue weight (8 weeks n=6; 16 weeks (chow n=8; HFD n=8); 28 weeks n=6; 51 weeks (chow n=7; HFD n=9); 83 weeks n=6). **(D)** Energy intake, food was weight before and after dietary intervention from each cage and divided to each individual mouse, assuming similar food intake per mouse (n represents cage with 3-4mice (8 weeks (chow n=4; HFD n=3); 16 weeks n=3; 28 weeks NA; 51 weeks n=3; 83 weeks n=2).Error bars represent mean ± s.e.m., Significances in B and C were calculated from chow and HFD combined groups, labeling for significances: from young (y), from adult (a), from middle age (m), from old (o), from very old (v), all are (P<0.0001) tested by One-way ANOVA + Tukey’s multiple comparison. If not indicated otherwise n denotes individual mice. eWAT, epididymal adipose tissue; EdU, 5-Ethynyl-2'-deoxyuridine.


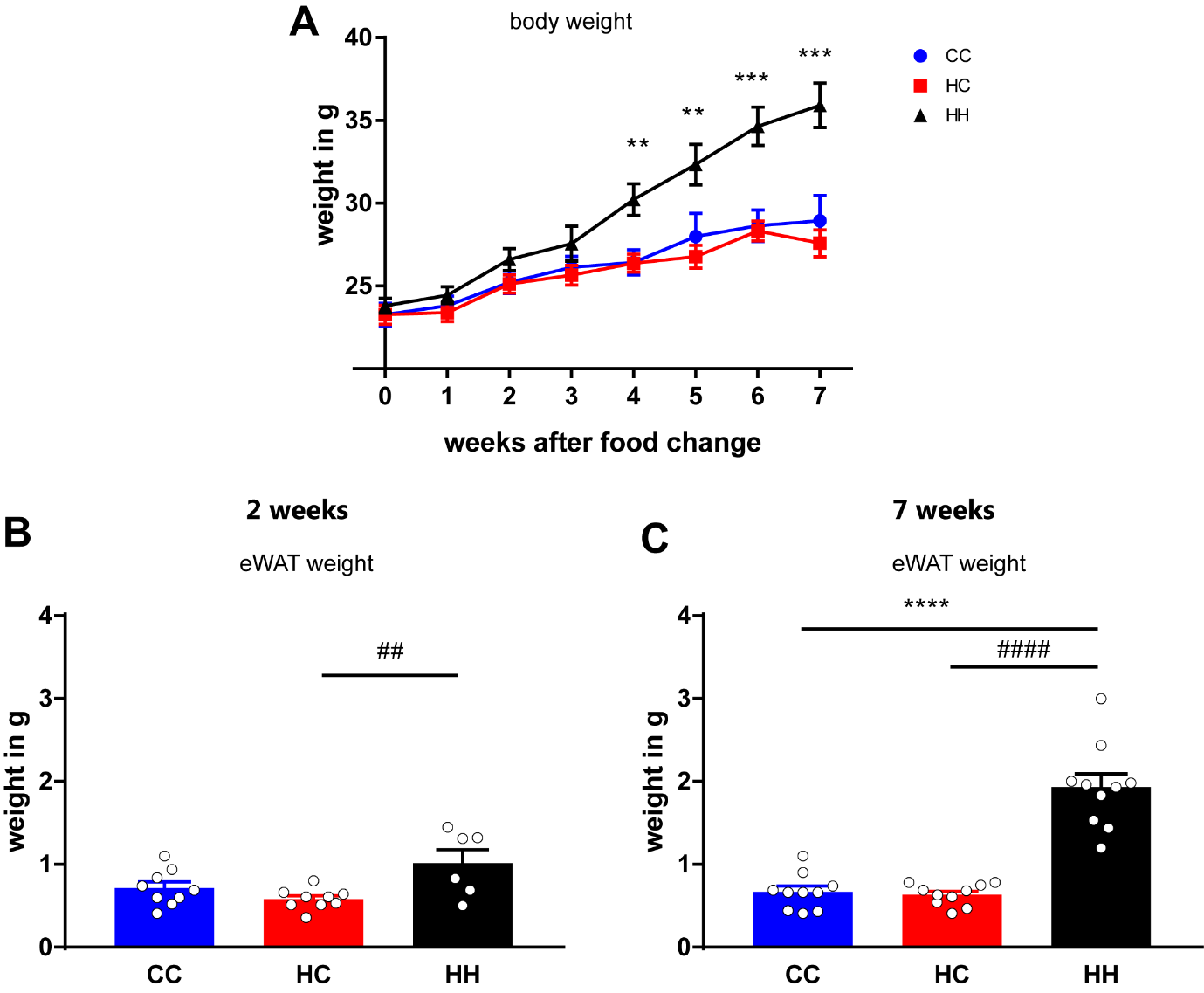


**Supplement figure 2**

**(A)** Body weight changes during the time course of the experiment in each treatment group (n=10). **(B-C)** eWAT tissue weight **(B)** 2 weeks (CC and HC n=9; HH n=6) and **(C)** 7 weeks (n=10 per group) after dietary intervention. Error bars represent mean ± s.e.m. ∗(P<0.05), ∗∗(P<0.01), ∗∗∗(P<0.001), ∗∗∗∗(P<0.0001) by **(A)** Two-way ANOVA + Tukey’s multiple comparison and **(B-C)** One-way ANOVA + Tukey’s multiple comparison. *=CC vs HH; # = HC vs HH. If not indicated otherwise n denotes individual mice. eWAT, epididymal adipose tissue.
